# Expression ratio of CCND1 to CDKN2A mRNA predicts RB1 status of cultured cancer cell lines and clinical tumor samples

**DOI:** 10.1186/1476-4598-10-31

**Published:** 2011-03-29

**Authors:** Shinji Mizuarai, Takumitsu Machida, Tsutomu Kobayashi, Hideya Komatani, Hiraku Itadani, Hidehito Kotani

**Affiliations:** 1 Departments of Oncology, Tsukuba Research Institute, Merck Research Laboratories, Banyu Pharmaceutical Co., Ltd., Tsukuba, Ibaraki 300-2611, Japan; 2 MSD K.K., Chiyoda-Ku, Tokyo 102-8667, Japan

## Abstract

**Background:**

The retinoblastoma product (RB1) is frequently deregulated in various types of tumors by mutation, deletion, or inactivation through association with viral oncoproteins. The functional loss of RB1 is recognized to be one of the hallmarks that differentiate cancer cells from normal cells. Many researchers are attempting to develop anti-tumor agents that are preferentially effective against RB1-negative tumors. However, to identify patients with RB1-negative cancers, it is imperative to develop predictive biomarkers to classify RB1-positive and -negative tumors.

**Results:**

Expression profiling of 30 cancer cell lines composed of 16 RB1-positive and 14 RB1-negative cancers was performed to find genes that are differentially expressed between the two groups, resulting in the identification of an RB1 signature with 194 genes. Among them, critical RB1 pathway components CDKN2A and CCND1 were included. We found that microarray data of the expression ratio of CCND1 and CDKN2A clearly distinguished the RB1 status of 30 cells lines. Measurement of the CCND1/CDKN2A mRNA expression ratio in additional cell lines by RT-PCR accurately predicted RB1 status (12/12 cells lines). The expression of CCND1/CDKN2A also correlated with RB1 status in xenograft tumors *in vivo*. Lastly, a CCND1/CDKN2A assay with clinical samples showed that uterine cervical and small cell lung cancers known to have a high prevalence of RB1-decifiency were predicted to be 100% RB1-negative, while uterine endometrial or gastric cancers were predicted to be 5-22% negative. All clinically normal tissues were 100% RB1-positive.

**Conclusions:**

We report here that the CCND1/CDKN2A mRNA expression ratio predicts the RB1 status of cell lines *in vitro *and xenograft tumors and clinical tumor samples *in vivo*. Given the high predictive accuracy and quantitative nature of the CCND1/CDKN2A expression assay, the assay could be utilized to stratify patients for anti-tumor agents with preferential effects on either RB1-positive or -negative tumors.

## Background

Retinoblastoma protein (RB1) is a crucial regulator of appropriate cell cycle progression, including G1 to S and G2 to M phase transitions [1,2]. The activity of RB1 is mainly regulated by the upstream CDKN2A/CCND1 pathway [3-5]. Anti-proliferative stresses including DNA damage, therapeutic agents, and anti-mitogens increase the expression of CDKN2A followed by the dissociation of CDK4 or CDK6/CCND1 complexes. The inactivation of CDKs/CCND1 leads to the hypophosphorylation of RB1, resulting in the transcriptional repression of E2F/DP regulatory genes that contributes to the progression of cell cycles including mini-chromosome maintenance (MCM) genes [6], A/D/E-type Cyclins [7-9], and CDC6 [10]. In accordance with the important role of RB1 as a cell cycle regulator, RB1 deregulation is frequently observed in multiple types of cancers [11]. Functional loss of RB1 causes accelerated E2F1 mediated transactivation, followed by uncontrolled cell cycle progression. In small cell lung cancer (SCLC), for example, the prevalence of RB1 loss is more than 90% [12,13], while RB1 function in cervical cancer is suppressed by directly associating with HPV-E7 oncoprotein at a frequency of at least 90% [14,15]. A moderate frequency of RB1 dysfunction has been reported in several other types of cancers such as bladder, prostate cancers, and melanoma [16-18]. The RB1 loss is caused by several molecular events such as point mutations, deletion, or inactivation through associating viral proteins [19]. In addition, genetic alteration or aberrant expression in upstream molecules also results in dysfunction of RB1. Accumulating evidence has shown that hypermethylation in the promoter region of CDKN2A or overexpression of CCND1, which results in RB1 dysfunction, frequently occurs in various types of cancers [11]. Therefore, deregulation in the RB1 pathway is recognized to be a hallmark of tumorigenesis.

Since most current standard-of-care anti-tumor drugs are cytotoxic agents, they do not possess selective killing effects on tumors compared with normal proliferating cells. Development of drugs which preferentially exert anti-tumor effects in tumors harboring specific deregulation in tumor-related genes is eagerly anticipated [20]. Given the different status of RB1 between tumor and normal cells, several studies have attempted to develop anti-tumor agents which selectively inhibit growth of tumors with dysfunctional RB1 [20-24]. The silencing of ECT2, which is a GTP exchanging factor involved in cytokinesis, is effective in RB1-negative cells when examined with RB1-positive and -negative isogenic cell lines in the presence of DNA damage [21]. It has also been shown that inhibition of Aurora B can induce increased apoptosis in RB1-deficient cells [22]. The response to standard chemotherapeutics is also affected by the status of RB1. RB1-negative cancers are more sensitive to some DNA-damaging agents such as 5-Fluorouracil [23], doxorubicin [24], and ionizing radiation [25], although the detailed mechanism for the RB1-negative selective effect remains unresolved. Tamoxifen is more effective against RB-positive breast cancers than -negative ones *in vitro *[26]. This knowledge might be integrated in future clinical trials to develop RB1-status dependent anti-tumor agents, and potentially increase the therapeutic window of those agents.

In order to utilize anti-tumor agents whose effect is different between RB1-positive and -negative cancers, the development of a predictive biomarker that discriminates the status of RB1 in cells is essential. Conventionally, the protein expression level of RB1 as measured by immunohistochemistry (IHC) has been used to estimate the prevalence of RB1 deficiency in various tumor types. Direct measurement of RB1 by IHC has generated vast amounts of information on RB1 deficiency in many types of cancers; however, it has several drawbacks as a biomarker to predict RB1 status. First, IHC for RB1 detects limited types of RB1 dysfunction, although RB1 loss can be caused by mutations, deletion, or association with viral proteins [11]. For instance, IHC for RB1 would be positive in tumors infected with HPV-virus expressing E7 oncoprotein, although RB1 is functionally lost [14]. Second, IHC is semi-quantitative, and comparisons of RB1 loss frequency among different studies are somewhat difficult [27]. This also makes it difficult to establish a threshold to distinguish RB-positive and -negative tumors that would guide investigators to determine whether the tumors be treated with anti-tumor agents whose effects are dependent on RB1 status [28]. Lastly, IHC methods mandate a relatively large amount of biopsy samples. When a predictive marker is used clinically, a minimally invasive biopsy is highly preferable from the patient's point of view. Therefore, the development of a novel RB1 predictive biomarker that overcomes these drawbacks would be useful for patient stratification by complementing or replacing the current direct RB1 protein measurement.

In the present study, we compared expression profiling of RB1-positive and -negative cell lines among 30 cancer cell lines, and identified a gene signature that was differentially expressed between the two groups. In the gene signature, CDKN2A and CCND1, which are the important nodal proteins in the RB1 pathway, were included. We found that the expression ratio of CCND1 to CDKN2A clearly classified RB1-positive and -negative cancer lines, the predictive accuracy of which was superior to that of individual CDKN2A or CCND1, respectively. The CCND1/CDKN2A ratio could also classify the RB1 status of xenograft tumors *in vivo*. Finally, CCND1/CDKN2A expressions were measured in formalin fixed paraffin embedded (FFPE) samples of clinical tumor and normal tissues. We found that uterine cervical and small cell lung cancers were predicted to be RB-negative at high prevalence, while most uterine endometrial and stomach cancers were predicted to be RB1-positive by the CCND1/CDKN2A assay.

## Results

### Identification of a gene signature to classify RB1 status by microarray analysis

We collected a panel of cancer cell lines to be used for the identification of a gene signature that discriminates between RB1-positive and -negative cell lines (Table 1). We first selected 11 cell lines, the RB1 status of which has been previously reported as negative. Other than the HeLa cell line, all the cell lines possess genetic alterations such as frame shifts or termination mutations, such that the function of RB1 is completely lost in 10 of the cell lines. Since the HeLa cell line expresses E7 proteins from papilloma virus, RB1 function is known to be dysfunctional. The RB1 functional status of the 11 cell lines and additional 19 cell lines was determined by a cellular assay which measures the activity of E2F-regulatory reporter gene in response to CDKN2A induction [21]. The cell lines were transfected with both the CDKN2A gene and the E2F regulatory reporter gene. In RB1-positive cells, the induced CDKN2A protein inhibits endogenous CDKs, leading to the activation of RB1 proteins, which is followed by the inhibition of E2F-mediated transcription. In RB1-negative cells, the E2F reporter gene does not respond to CDKN2A induction. With the cellular RB1 functional assay, 16 cell lines were identified as RB1-positive because they showed significant inhibition of the E2F reporter gene upon induction of the CDKN2A protein, while the other three cell lines were characterized as RB1-negative (Additional file 1A). In total, 16 RB1-positive and 14 RB1-negative cell lines were used for the identification of an RB1 classifier (Table 1).

**Table 1:** RB1 status of 30 cancer cell lines used for gene expression

Cell line	Tissue type	RB1 genetic status	RB1 functional status
T-24	bladder	wild-type	+
U-2-OS	bone	wild-type	+
SF268	CNS	wild-type	+
DLD-1	colon	NA	+
HCT-116	colon	wild-type	+
Hep G2	liver	NA	+
NCI-H460	NSCLC	wild-type	+
A549	NSCLC	wild-type	+
UACC-62	melanoma	wild-type	+
MDAH-2774	ovary	NA	+
PANC-1	pancreas	NA	+
SU.86.86	pancreas	NA	+
NCI-N87	stomach	wild-type	+
Hs 746T	stomach	NA	+
KATOIII	stomach	wild-type	+
SCC-25	tongue	wild-type	+
J82	tongue	Δ21 2107-2A > G	-
HeLa S3	bladder	repressed by E7	-
DU-145	cervix	stop: K175*	-
TCCSUP	bladder	deletion: 1696_2787del1092	-
MDA-MB-436	breast	frame shift: G203fs*8	-
MDA-MB-468	breast	deletion: 265_2787del2523	-
SF-539	CNS	deletion: T116fs*8	-
HeLa	cervix	repressed by E7	-
C-33A	cervix	deletion: 4aa in exon2	-
Lu-135	SCLC	frame shift: R661fs*1	-
NCI-H128	SCLC	frame shift: R418fs*2	-
NCI-H1417	SCLC	stop: Y321*	-
NCI-H69	SCLC	stop: E748*	-
NCI-H596	NSCLC	frame shift: R661fs*1	-

*The functional assay which determined the status of RB1 in the present study is described in Methods and data are shown in Additional file 1.

**Figure 1:**
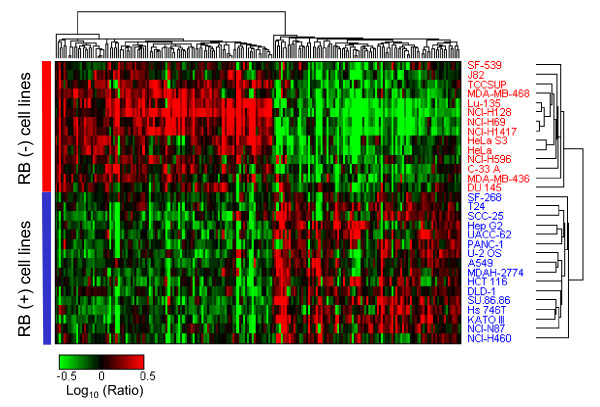
**Identification of a gene expression signature to classify RB1 status**. The expression profiles of 16 RB1-positive and 14 RB1-negative cancer cell lines were analyzed. One hundred and ninety-four genes which were differentially expressed between the two groups were extracted as described in Materials and Methods. For the selected genes and 30 cell lines, two-dimensional hierarchical clustering was conducted. Each row represents a cell line. Each column represents a gene. Red, up-regulated genes; green, down-regulated genes.

Next, to find genes which are differentially expressed between the RB1-positive and -negative cell lines, mRNA expression profiles were carried out with an Agilent oligonucleotide microarray. All microarray hybridization was performed with Human Universal Reference RNAs to enable comparative analysis among the cell lines. We selected genes whose standard deviation of expressional log_10 _ratio in all 30 cell lines was greater than or equal to 0.1. We further extracted genes whose p-value of Pearson correlation coefficiency between expressional log_10 _ratio and RB1 status was less than or equal to 0.001. As a result, 194 genes were selected as an RB1 status signature. When hierarchical 2 D clustering was performed with the genes and the 30 cell lines, the 16 RB1-positive and 14 RB1-negative cell lines were accurately classified into the two groups (Figure 1), suggesting that the gene signature could be a tool to determine the status of RB1. The signature included RB1 itself and its expression in the 30 cell lines are shown in Additional file 1B. When a hypergeometric test for gene enrichment was conducted against the RB1 signature gene, the examination showed that the genes whose function is related to the G1-S phases of cell cycle were significantly condensed in up-regulated genes (G1 phase: 5.9 × 10^-3^; S phase: 4.2 × 10^-2^). The results of the enrichment test were in accordance with the function of RB1 as a cell cycle regulator through G1 to S phase transition.

### Expression ratio of CCND1 to CDKN2A predicts RB1 status

**Figure 2:**
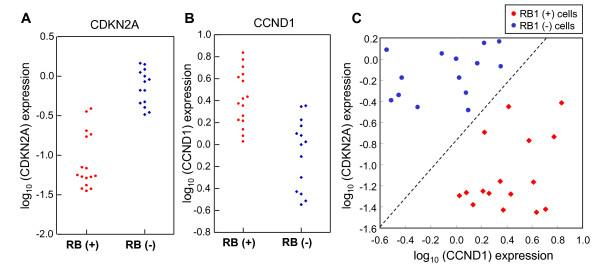
**Expression pattern of CCND1 and CDKN2A in RB-positive and -negative cell lines**. Correlation between RB1 status and CDKN2A (A) or CCND1 (B) expression level derived from microarray data. **(C) **Relationship between RB1 status and the ratio of CCND1 to CDKN2A expression derived from microarray data.

An RB1 classifier would be useful as a predictive biomarker for some anti-tumor agents whose efficacy depends on RB1 status. In order to use an RB1 classifier in clinical trials, it is highly preferable to reduce the number of genes without affecting the prediction accuracy. Within the RB1 gene signature composed of 194 genes, we found that CDKN2A and CCND1, which are key regulators in the RB1 pathway, were included. When we compared the RB1 status with either CDKN2A or CCND1 expression, both of them were highly correlated (p < 0.01). This is in accordance with previous reports that RB1 status is inversely correlated with CDKN2A [13,14]. However, the expression level of either CDKN2A or CCND1 alone did not accurately classify the 30 RB1-positive or -negative cell lines that were used to find the RB1 gene signature (Figure 2A and 2B). We next explored whether a combination of CCND1 and CDKN2A expression data would predict RB1 status. In Figure 2C, the correlation of CDKN2A and CCND1 expressions and RB1 status is shown. We found that the expression ratio of CCND1 and CDKN2A precisely classified the RB1-positive and -negative cell lines.

### Validation of the CDKN2A and CCND1 ratio as an RB1 classifier

**Figure 3:**
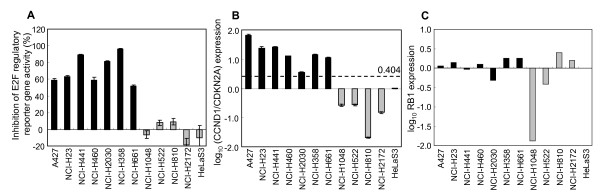
**CCND1/CDKN2A expression predicts RB1 status**. **(A) **RB1 status of 12 cell lines determined by RB1 functional assay. Each cell line was transfected with E2F-regulatory reporter SEAP plasmid with or without the CDKN2A expression vector. The inhibition level of the SEAP reporter gene activity in response to CDKN2A induction was normalized to luciferase activity. **(B) **RB1 status of 12 cell lines predicted by the expression ratio of CCND1/CDKN2A. mRNAs from the 12 cell lines were analyzed with quantitative RT-PCR analysis for CCND1 and CDKN2A. The threshold of CCND1/CDKN2A to determine RB1 status was established as 0.404 by discriminate analysis. The RB1 statuses determined by CCND1/CDKN2A coincided with those determined by RB1 function assay. **(C) **Relative RB1 mRNA expression level in 12 cell lines measured with quantitative RT-PCR assay.

**Figure 4:**
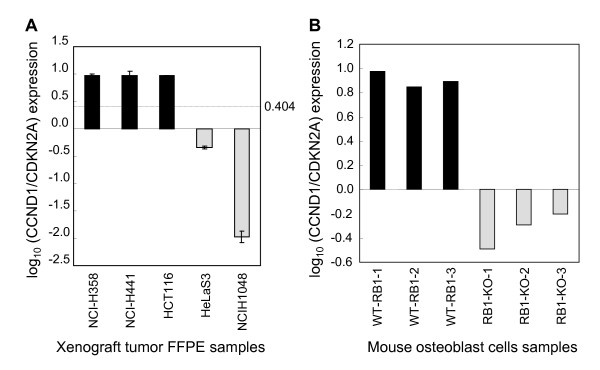
**CCND1/CDKN2A expression predicts RB1 status in animal tumor models**. **(A) **mRNAs from five xenograft tumors were purified from FFPET and analyzed with quantitative RT-PCR for CCND1 and CDKN2A. NCI-H358, NCI-H441, and HCT116 cell lines are known to be RB1-positve. HeLaS3 and H1048 cell lines are known to be RB1-negative. The cut-off value of log_10 _(CCND1/CDKN2A) ratio to classify RB1 status is shown: 0.404. **(B) **CCND1/CDKN2A expression was analyzed in osteoblast cells derived from RB1 wild-type and RB1 knockout mice using publicly available microarray expression data (GEO: GSE19299).

The predictive accuracy of RB1 status by the CCND1/CDKN2A ratio was examined with the original 30 cancer cell lines which were used to develop the RB1 gene signature as a training set (Figure 2B). We next investigated whether the CCND1/CDKN2A expression ratio could predict the RB1 status of an additional 10 cell lines (Figure 3A) in which the RB1 status was unknown, and with HeLaS3 and H460 cells as controls of RB-negative and -positive cell lines. The functional RB1 status of the 12 cell lines was determined by the CDKN2A/E2F reporter gene assay, resulting in seven and five cell lines as RB1-positive and -negative, respectively (Figure 3A). Next, CCND1 and CDKN2A expressions were determined by quantitative RT-PCR. The expression ratio of CCND1/CDKN2A clearly classified the RB-positive and -negative groups determined by the RB1 functional assay (Figure 3B), although RB1 mRNA expression level were not associated with the functional RB1 status (Figure 3C). Discriminant analysis with the CCND1 to CDKN2A expression ratio data generated by quantitative RT-PCR from the 12 cell lines established 0.404 as the cut-off value of log_10 _(CCND1/CDKN2A) ratio to classify RB1 status, which is detailed in Materials and Methods. Within the 12 test cell lines, the expression ratio of CCND1 to CDKN2A predicted RB1 status with an accuracy of 100%. Next, to examine whether CCND1 and CDKN2A could be utilized as an RB1 classifier *in vivo*, the RB1 status was determined in xenograft tumor samples. The reasons to study xenograft tumors are: 1) to examine mRNA samples from formalin fixed paraffin embedded tissues (FFPET), it is useful to predict RB1 status by the CCND1/CDKN2A expression assay; 2) to examine whether three-dimensional structures in xenograft tumors do not affect the expression ratio. To develop xenograft tumors, cancer cells were inoculated to nude rats, and xenograft tumors were formed for 1-2 weeks. The developed xenograft tumors were fixed in 10% formalin and stored as FFPET. We extracted mRNA from the FFPET samples, and performed quantitative real-time RT-PCR. As shown in Figure 4, CCND1/CDKN2A expression ratios determined with mRNA extracted from FFPET were almost equivalent to those determined from mRNA extracted from tissue cultures *in vitro*, and predicted three xenograft tumors as RB1-positive and the other two as RB1-negative, which was 100% accurate. This implies that the RB1 classifier using the CCND1/CDKN2A ratio would apply to FFPET samples *in vivo*. We further validated the RB1 classifier with two publicly available microarray data sets. First, we applied the RB1 classifier to RB1 wild-type and RB1 knockout mouse osteoblast samples, and CCND1/CDKN2A expression ratio clearly discriminated the two genotypes (Figure 4B). Second, expression profiling data of RB1-expressiong and RB1-deficient paired SAOS-2 cell lines were analyzed. The CCDN1/CDKN2A were significantly different between the two isogenic cells (Additional file 2).

### Validation of CCND1/CDKN2A expression in clinical samples

**Figure 5:**
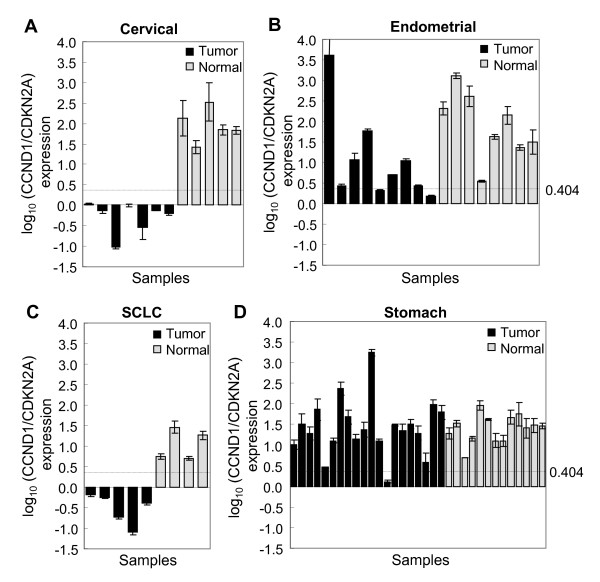
**CCND1/CDKN2A expression in clinical tumor and normal tissues**. FFPETs of clinical samples were obtained from the Kanagawa Cancer Research and information Association. mRNAs from normal and tumor tissues were purified and analyzed with quantitative RT-PCR for CCND1 and CDKN2A. The tissue type is designated in each figure; **(A) **cervical, **(B) **endometrial, **(C) **small cell lung, **(D) **stomach cancers and corresponding normal tissues. The cut-off value of log_10 _(CCND1/CDKN2A) ratio to classify RB1 status is shown: 0.404.

To examine whether the CCND1/CDKN2A expression ratio predicts RB1 status in clinical tumor samples, human clinical tumor and normal samples were analyzed in CCND1/CDKN2A assays. Uterine cervical, endometrial, small cell lung, stomach cancers, and their corresponding normal tissues were investigated. RNA was extracted from FFPET of the clinical samples and subjected to quantitative RT-PCR. As shown in Figure 5, the CCND1/CDKN2A ratios of all the normal tissues were significantly higher compared with those of corresponding tumor tissues, indicating that the RB1 status of all the normal tissues was positive. Cervical cancer and SCLC, which are known to be the tumor types with a higher frequency of dysfunctional RB1, showed lower CCND1/CDKN2A ratios compared with endometrial and gastric cancers. The predicted RB-negative frequencies determined by the cut-off value of log_10 _(CCND1/CDKN2A) of 0.404 were 100% (7/7), 22% (2/9), 100% (5/5), and 5% (1/20) in cervical, endometrial, small cell lung, and gastric cancers, respectively (Table 2). All the normal samples from the four tissues were predicted to be RB-positive (100%) by the cut-off value. Since measuring RB1 expression is a current standard method to determine RB1 status in clinical samples, we analyzed RB1 and CCND1/CDKN2A mRNA expression levels in 6 SCLC and 17 normal lung samples using publicly available microarray data (Additional file 3). The CCND1/CDKN2A ratio was significantly lower in all the SCLC samples compared with normal lung samples, which is in accordance with our data.

**Table 2:** RB1-negative frequency in clinical samples determined by the CCND1/CDKN2A expression ratio

Tissue	Cervical	Endometrial	SCLC	Stomach
Normal	0% (0/5)	0% (0/7)	0%	0% (0/13)
Tumor	100% (7/7)	22% (2/9)	100% (5/5)	5% (1/20)

## Discussion

Deregulation of RB1 has been reported in multiple types of tumors, including retinoblastoma, cervical cancer, and SCLC. Development of anti-tumor agents which selectively kill RB1-negative tumors is expected to increase the therapeutic window for treatment of tumors with an RB1-negative background. Hence, various researchers are trying to develop novel anti-tumor agents with selective anti-tumor effects on RB1-deficient tumors [20,22,24], or find RB1 context specificity for current standard-of-care medicines [23,24]. Examples include the studies of topoisomerase inhibitors or ECT2 inhibition which are more effective in RB1-negative tumors compared with RB1-positive counterparts [20,21]. Since topoisomerases or ECT2 are regulated by E2F transcription factors as downstream effectors of RB1, it is reasonable that their inhibitions are sensitive to RB1-negative tumors. In order to apply the information of the RB1 context specificity to clinical trials, it is necessary to develop a classifier of RB1 status of clinical samples. The CCND1/CDKN2A assay as a predictive biomarker for RB1 demonstrated in the present study has several advantages. First, the assay showed 100% prediction accuracy in the original 30 cancer cell lines determined by microarray, and 12 cultured cell lines and four xenograft tumors determined by quantitative qPCR. The prevalence of RB1 deficiency in clinical tumor and normal samples were also consistent with previous reports using IHC for RB1. Second, the assay provides quantitative and reproducible data when determined by RT-PCR with defined primer and probe sequences. Therefore, the cut-off value to discriminate RB1-positive and -negative cell lines by discriminant analysis could be established, while criteria to determine RB1 prevalence by conventional IHC-based assays is relatively arbitrary. Third, although RB1 protein can be expressed as an inactive form when tumor cells harbour missense mutation in RB1 gene or HPV-mediated RB1 inactivation which makes it difficult for IHC to classify RB1 functional status, the CCND1/CDKN2A ratio expression assay could detect various types of RB1 functional inactivation as shown in Figure 1 and 2. Finally, the RT-PCR assay requires a smaller amount of biopsy specimen. Recent advances in RNA/cDNA amplification might enable investigators to conduct the assay with less than 10 cells, in contrast to IHC which mandates several slices of FFPET with good morphological integrity.

An inverse correlation of CDKN2A and RB1 expressions has been reported in several types of cancers [12,13]. In cervical cancers infected with HPV, the function of RB1 is lost due to association with the E7 oncoprotein. Cervical cancers express high levels of CDKN2A probably by a feedback mechanism [14]. An anti-correlation of RB1 and CDKN2A expression was also reported in SCLC, gastric cancer, oral cavity squamous carcinoma or urinary bladder cancer [13,29-31]. In our experiments, the expression levels of both CDKN2A and CCND1 were negatively and positively correlated with RB1 status in accordance with previous reports; however, neither accurately classified the status of RB1 as demonstrated in Figure 2A. By combining the expressions of CCND1 and CDKN2A, the prediction accuracy was significantly increased to 100% in all 30 samples in the microarray, and in an additional study with 12 cell lines in RT-PCR analysis.

Numerous studies with expression profiles have reported gene signatures which are differentially expressed between RB1-positive and -negative cells [32-35]. Markey et al. found that more than 200 genes are decreased by the induction of a mutated RB1 that is refractory to CDK-mediated phosphorylation [32]. The function of the gene signature is related to cell cycle control, DNA repair, or chromatin control, consistent with the well-established role of RB1. Semizarov et al. reported another set of genes that are commonly deregulated by several different sequences of siRNA for RB1 [33]. Pathway analysis of the gene set by GeneMapp and MappFinder software packages found that cellular processes involved in DNA replication, mitotic cell cycle, and chromosome maintenance were statistically condensed in the gene set. These pioneering works analyzing global expression profiling of genetically engineered RB1 loss highlighted the downstream effectors, which are responsible for the function of RB1 to control cell cycle or tumorigenesis. We tested whether the previously reported RB1 regulated gene signatures could classify the RB1-positve and -negative cell lines used in the present study (Table 1); however, the signatures did not distinguish the RB1 status of the cell lines (data not shown). The present study provides an RB1 classifier that distinguishes the status of RB1 in both cell lines and clinical samples. Since expression profiling of naturally occurring RB1-positive and -negative cell lines was used to develop the signature followed by the narrowing-down of genes with biological information, this may have resulted in higher accuracy for predicting RB1 status in additional cell lines and xenograft tumors.

The prevalence of RB1 (-) in clinical tumors and normal samples was reasonable compared with previous reports using IHC. While several reports have shown that loss of RB1 is found in more than 90% of clinical SCLCs [12], 100% (5/5) of SCLC samples examined here were RB1-negative when classified by the CCND1/CDKN2A expression assay. The reported RB1 loss frequency of uterine cervical cancers and uterine endometrial cancers is more than 90% [14,15] and 3-6% [36,37], respectively. Our assay showed 100% (7/7) and 22% (2/9) for cervical and endometrial cancers, respectively. The prevalence of RB1 (-) in previous studies of gastric cancers using IHC for RB1 is relatively variable, ranging from 0-40% [27,28,38,39] depending on the different criteria used among the studies. The CCND1/CDKN2A assay predicted 5% (1/20) as RB1 (-) cancers. In general, the frequency of RB1 loss is highly correlated between previous reports and the present study, and the data imply that the CCND1/CDKN2A assay could be utilized to predict RB1 status clinically as a quantitative assay.

Development of predictive biomarkers is of great significance for anti-tumor agents, since the response rate is relatively lower compared with other diseases. In order to develop a clinically available gene signature as a biomarker, it is preferable to develop a biomarker composed of a minimal number of genes [40]. Although microarray technology allows us to measure genome-wide expression profiles without limiting the number of genes, it would be difficult to use the platform to stratify patients in a clinical setting from the viewpoints of cost performance and assay turn-over. A quantitative RT-PCR-based method with two genes would be feasible for conventional clinical trials. Additionally, a PCR-based method to measure the status of RB1 as a biomarker might be developed as an *in vitro *diagnostic (IVD), since some IVDs utilize PCR to detect biomarkers such as Ras, EGFR mutation, or HIV infection [41,42].

## Conclusions

The present study identified that the expression ratio of CCND1/CDKN2A predicts the status of RB1 in both cultured cancer cell lines and clinical tumor samples. Since many studies have been attempting to find currently available or novel anti-tumor agents which are selectively effective for tumors with dysfunctional RB1, the CCND1/CDKN2A ratio expression assay might contribute to patient stratification as a highly accurate, quantitative, and less invasive biomarker.

## Methods

### Cell lines and RB1 functional assay

Cell lines used to detect RB1 status listed in Table 1 were obtained from the American Type Culture Collection (ATCC), and were cultured according to the supplier's instructions. The RB1 functional status of each cell line was determined by measuring the activity of the E2F-regulatory reporter gene in response to CDKN2A induction [20]. Cells in 6-well plates were co-transfected with 100 ng of CDKN2A expression plasmid [20], 100 ng of luciferase reporter plasmid (pGL3-SV40, Promega, Madison, WI) for normalization of transfection efficiency, and 100 ng of SEAP reporter plasmid containing the E2F-responsive element, pSEAP2-CDC6 [20] by a lipofection method using FuGENE6 (Roche Diagnostics, Mannheim, Germany). At 48 hr post transfection, SEAP activity was measured with a Reporter Assay Kit-SEAP (Tokyo, Osaka, Japan). The SEAP activity was normalized to luciferase activity. RB1 mutation information of cell lines were obtained from the literature (Table 1) and from the COSMIC database at Sanger Center (URL: http://www.sanger.ac.uk/genetics/CGP/cosmic/).

### Expression profiling of cell lines

Total RNAs were extracted from cells growing in log phase using an RNeasy Kit (QIAGEN, Valentia, CA), and were subjected to mRNA profiling using custom Human 3.0 A1 arrays (Agilent Technologies, Santa Clara, California) as directed by the manufacturer's protocol. As a common reference sample for all cell lines, Human Universal Reference RNA (Stratagene, La Jolla, CA) was used. Scanned data were processed by Resolver (Rosetta Biosoftware, Seattle, WA) to calculate the ratios and p-values based on an error-weighted model for each probe. For statistical analysis, R (GNU freeware) and MATLAB (MathWorks Inc., Natick, MA) were used. To identify genes whose expressions were correlated with RB1 status, we first filtered out genes whose standard deviation of log ratio values were less than 0.1 to exclude genes with poor expression changes among cell lines, and selected 194 genes whose expression levels showed a p-value of 0.001 or less for Pearson correlation coefficient with RB1 status (1 for RB1 positive, 0 for RB1 negative).

### Quantitative RT-PCR analysis

cDNA was synthesized from 1 μg of total RNA by using TaqMan reverse transcription reagents (PE Applied Biosystems, #N8080234). Quantitative real-time PCR assays for human CCND1, CDKN2A and RB1 were performed in triplicate for cDNA samples in 384-well optical plates. Data were collected and analyzed using an ABI PRISM 7900 sequence detector system (Applied Biosystems, Foster City, CA). Pre-designed TaqMan probes and primers for CCND1 (Hs00277039_m1), CDKN2A (Hs00233365_m1) and RB1 (Hs00153108_m1) genes were purchased from Applied Biosystems, Inc. The CCND1:CDKN2A ratio was calculated as follows:

For prediction of RB1 status, the log10 (CCND1/CDKN2A) data of the test cell lines which include 7 RB1-positive and 5 RB1-negative lines were obtained by the quantitative RT-PCR. Then, the following linear discriminant function was derived: Y = 0.54 × log10 (CCND1/CDKN2A)-2.051094.

Then the functional RB1 status was determined as follows:

Y > 0 (log10 (CCND1/CDKN2A) > 0.404), positive functional RB1 status; and

Y < 0 (log10(CCND1/CDKN2A) < 0.404) negative functional RB1 status.

### Xenograft tumor samples

All animal studies were carried out in accordance with good animal practices as defined by the Institutional Animal Care and Use Committee (IACUC). Tumor cells (NCI-H353, NCI-H441, HCT116, HeLaS3, and NCI-H1048) were inoculated in the hind flank of immunodeficient nude rats (F344/NJcl-rnu, CLEA Japan) as a suspension in Matrigel (BD Bioscience, San Jose, CA). After 1-2 weeks, xenograft tumors were collected and stored as FFPETs. Extraction of RNA from normal and tumor FFPET samples was carried out using FFPE-RNeasy kit (QIAGEN). The purified RNA was subjected to CCND1/CDKN2A expression analysis.

### Clinical human tumors and normal tissues

Cervical, endometrial, gastric, small cell lung cancers and corresponding normal tissue samples with informed consent were obtained from Kanagawa Cancer Research and Information Association (Kanagawa, Japan). Individual institutional ethical committees approved the use of all clinical materials. FFPET of the tumors were used for RNA purification. First, normal tissues were dissected and collected from a section of FFPET if the section contained enough normal tissue. The tumor samples contained small amount of normal samples by the macro-dissection. Extraction of RNA from the normal and tumor FFPET samples was carried out using a FFPE-RNeasy kit (QIAGEN). The purified RNA was subjected to CCND1/CDKN2A expression analysis.

### Microarray analysis of public data

Expression profiling data for mouse osteoblast cells derived from both RB1 wild-type and RB1 deficient mice were retrieved from the NCBI Gene Expression Omnibus (GEO: GSE19299). Expression profiling data for RB1-expressing and RB1-deficient SAOS-2 paired cell lines were also retrieved from the NCBI GEO (GSE9690). Expression profiling data of SCLC and normal lung samples were retrieved from Broad Institute website http://www.broadinstitute.org/mpr/lung/. The signal intensities of the probe sets were normalized using the RMA method, and log ratio values to the average of all of the samples were calculated. The log10(CCDN1/CDKN2A) value was then calculated for each data.

## Competing interests

The authors declare that they have no competing interests.

## Authors' contributions

SM contributed to the conception, design, statistical data analysis and manuscript preparation. TM performed gene expression studies of preclinical samples. TK conducted gene expression studies of clinical samples. HK participated in the design, supervision of the study. HI performed microarray data analysis. Hito K contributed to the design, coordination, and guidance of the study. All authors read and approved the final manuscript.

## Supplementary Material

Additional file 1 **RB1 status of 30 cell lines determined by RB1 functional assay**. **(A) **Each cell line was transfected with E2F-regulatory reporter SEAP plasmid with or without the CDKN2A expression vector. The inhibition level of the SEAP reporter gene activity in response to CDKN2A induction was normalized to luciferase activity. **(B) **RB1 mRNA expression level measured by microarray. Relative mRNA expressions of 30 cell lines were shown as log10 ratio to HeLaS3 cells. Black bar: functionally RB-positive cells; gray bar: functionally RB-negative cells. Click here for file

Additional file 2 **CCND1/CDKN2A expression distinguishes RB1-expressing and RB1-deficient SAOS-2 cells**. CCND1/CDKN2A expression ratio was analyzed in both RB1-expressing and RB1-deficient SAOS-2 cells using publicly available microarray expression profiling data (GEO: GSE9690). Click here for file

Additional file 3 **CCND1/CDKN2A expression in clinical tumor and normal tissues**. CCND1/CDKN2A expression ratio **(A) **and RB1 expression **(B) **in SCLC and normal lung samples were analyzed using publicly available tumor microarray data that was retrieved from online supplement data at http://www.genome.wi.mit.edu/MPR/lung. Click here for file
