# Supplementary material for: Expression ratio of CCND1 to CDKN2A mRNA predicts RB1 status of cultured cancer cell lines and clinical tumor samples
Source: Mol Cancer. 2011 Mar 29;10:31. doi: 10.1186/1476-4598-10-31 (PMC3072353; doi:10.1186/1476-4598-10-31)
Supplement: Additional file 1 — RB1 status of 30 cell lines determined by RB1 functional assay. (A) Each cell line was transfected with E2F-regulatory reporter SEAP plasmid with or without the CDKN2A expression vector. The inhibition level of the SEAP reporter gene activity in response to CDKN2A induction was normalized to luciferase activity. (B) RB1 mRNA expression level measured by microarray. Relative mRNA expressions of 30 cell lines were shown as log10 ratio to HeLaS3 cells. Black bar: functionally RB-positive cells; gray bar: functionally RB-negative cells. [file 1476-4598-10-31-S1.PPT]

## Slide 1
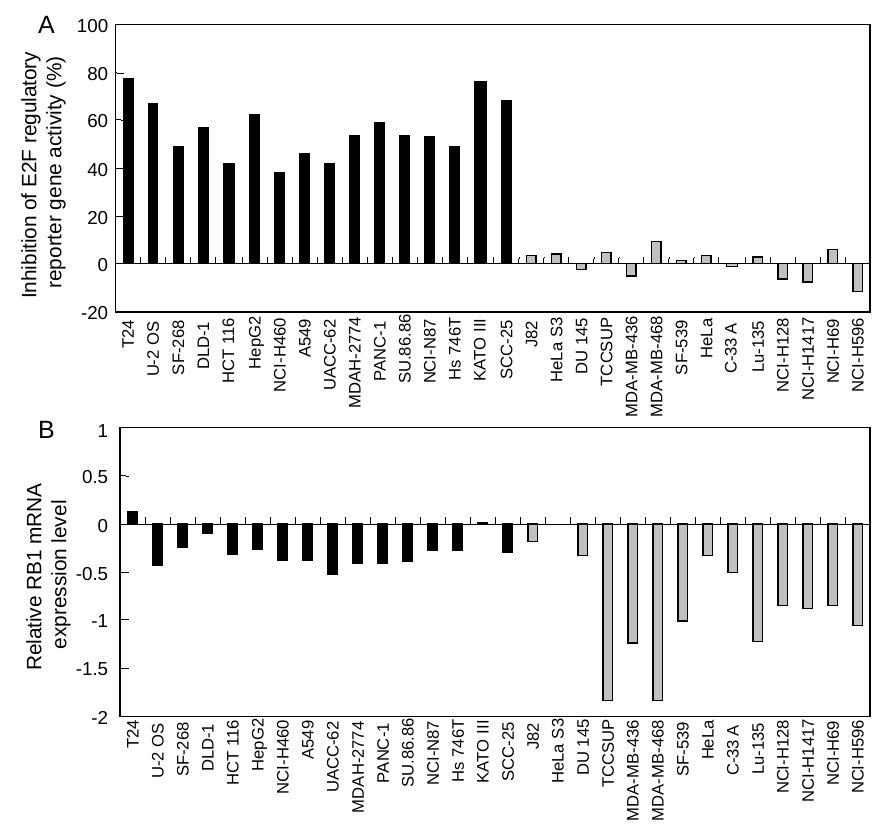

A
100
80
60
Inhibition of E2F regulatory
reporter gene activity (%)
40
20
0
-20
J82
T24
A549
HeLa
HepG2
DLD-1
DU 145
Lu-135
SF-268
SF-539
C-33 A
U-2 OS
Hs 746T
SU.86.86
HeLa S3
KATO III
SCC-25
HCT 116
PANC-1
NCI-N87
NCI-H69
TCCSUP
NCI-H460
NCI-H128
NCI-H596
UACC-62
NCI-H1417
MDAH-2774
MDA-MB-436
MDA-MB-468
B
1
0.5
0
Relative RB1 mRNA
expression level
-0.5
-1
-1.5
-2
T24
J82
HeLa
A549
HepG2
DU 145
DLD-1
Lu-135
SF-539
SF-268
C-33 A
U-2 OS
Hs 746T
HeLa S3
KATO III
SCC-25
HCT 116
PANC-1
SU.86.86
NCI-N87
TCCSUP
NCI-H69
NCI-H128
NCI-H596
UACC-62
NCI-H460
NCI-H1417
MDAH-2774
MDA-MB-436
MDA-MB-468
