# Supplementary material for: Expression ratio of CCND1 to CDKN2A mRNA predicts RB1 status of cultured cancer cell lines and clinical tumor samples
Source: Mol Cancer. 2011 Mar 29;10:31. doi: 10.1186/1476-4598-10-31 (PMC3072353; doi:10.1186/1476-4598-10-31)
Supplement: Additional file 2 — CCND1/CDKN2A expression distinguishes RB1-expressing and RB1-deficient SAOS-2 cells. CCND1/CDKN2A expression ratio was analyzed in both RB1-expressing and RB1-deficient SAOS-2 cells using publicly available microarray expression profiling data (GEO: GSE9690). [file 1476-4598-10-31-S2.PPT]

## Slide 1
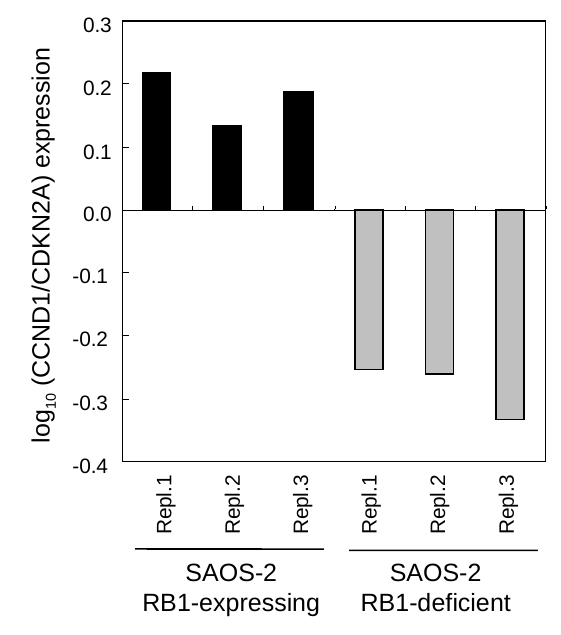

0.3
0.2
0.1
0.0
log10 (CCND1/CDKN2A) expression
-0.1
-0.2
-0.3
-0.4
Repl.1
Repl.2
Repl.3
Repl.1
Repl.2
Repl.3
SAOS-2
RB1-expressing
SAOS-2
RB1-deficient
