# Supplementary material for: Expression ratio of CCND1 to CDKN2A mRNA predicts RB1 status of cultured cancer cell lines and clinical tumor samples
Source: Mol Cancer. 2011 Mar 29;10:31. doi: 10.1186/1476-4598-10-31 (PMC3072353; doi:10.1186/1476-4598-10-31)
Supplement: Additional file 3 — CCND1/CDKN2A expression in clinical tumor and normal tissues. CCND1/CDKN2A expression ratio (A) and RB1 expression (B) in SCLC and normal lung samples were analyzed using publicly available tumor microarray data that was retrieved from online supplement data at http://www.genome.wi.mit.edu/MPR/lung. [file 1476-4598-10-31-S3.PPT]

## Slide 1
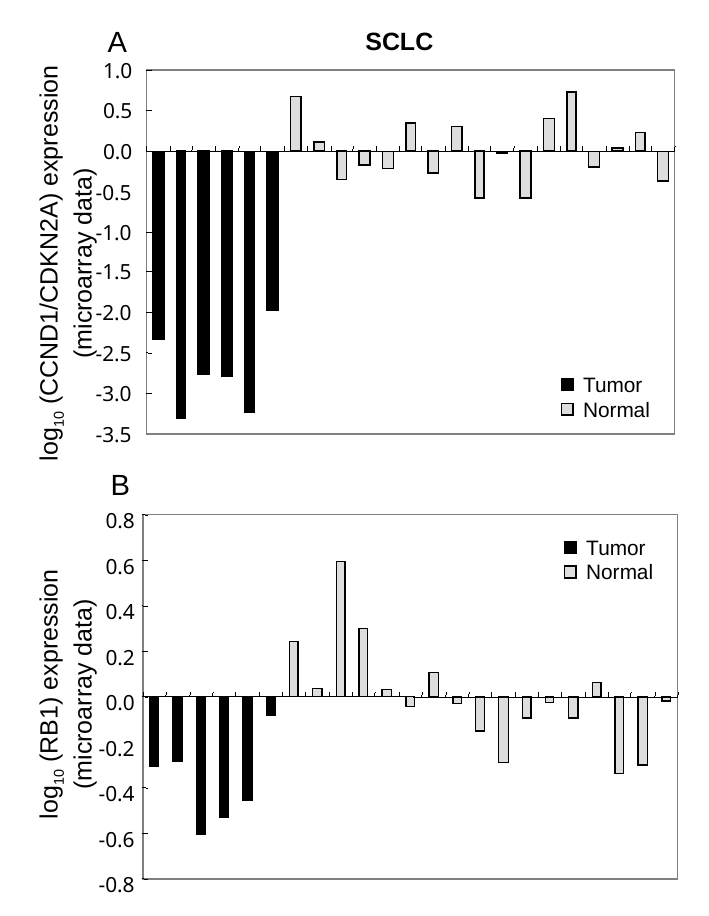

A
SCLC
1.0
0.5
0.0
-0.5
-1.0
log10 (CCND1/CDKN2A) expression
(microarray data)
-1.5
-2.0
-2.5
Tumor
Normal
-3.0
-3.5
B
0.8
Tumor
Normal
0.6
0.4
0.2
log10 (RB1) expression
(microarray data)
0.0
-0.2
-0.4
-0.6
-0.8
